# Posttraumatic and Idiopathic Spike–Wave Discharges in Rats: Discrimination by Morphology and Thalamus Involvement

**DOI:** 10.3390/neurolint15020038

**Published:** 2023-04-27

**Authors:** Ilia Komoltsev, Olga Salyp, Aleksandra Volkova, Daria Bashkatova, Natalia Shirobokova, Stepan Frankevich, Daria Shalneva, Olga Kostyunina, Olesya Chizhova, Pavel Kostrukov, Margarita Novikova, Natalia Gulyaeva

**Affiliations:** 1 Department of Functional Biochemistry of the Nervous System, Institute of Higher Nervous Activity and Neurophysiology, Russian Academy of Sciences, Moscow 117485, Russia; 2 Moscow Research and Clinical Center for Neuropsychiatry, Moscow 115419, Russia

**Keywords:** epilepsy, spike–wave discharge, thalamus, EEG, traumatic brain injury, epileptogenesis

## Abstract

The possibility of epileptiform activity generation by the thalamocortical neuronal network after focal brain injuries, including traumatic brain injury (TBI), is actively debated. Presumably, posttraumatic spike–wave discharges (SWDs) involve a cortico-thalamocortical neuronal network. Differentiation of posttraumatic and idiopathic (i.e., spontaneously generated) SWDs is imperative for understanding posttraumatic epileptogenic mechanisms. Experiments were performed on male Sprague-Dawley rats with electrodes implanted into the somatosensory cortex and the thalamic ventral posterolateral nucleus. Local field potentials were recorded for 7 days before and 7 days after TBI (lateral fluid percussion injury, 2.5 atm). The morphology of 365 SWDs (89 idiopathic before craniotomy, and 262 posttraumatic that appeared only after TBI) and their appearance in the thalamus were analyzed. The occurrence of SWDs in the thalamus determined their spike–wave form and bilateral lateralization in the neocortex. Posttraumatic discharges were characterized by more “mature” characteristics as compared to spontaneously generated discharges: higher proportions of bilateral spreading, well-defined spike–wave form, and thalamus involvement. Based on SWD parameters, the etiology could be established with an accuracy of 75% (AUC 0.79). Our results support the hypothesis that the formation of posttraumatic SWDs involves a cortico-thalamocortical neuronal network. The results form a basis for further research of mechanisms associated with posttraumatic epileptiform activity and epileptogenesis.

## 1. Introduction

Epilepsy is one of the most common neurological diseases; it affects more than 70 million people worldwide. Traumatic brain injury (TBI) is among the most frequent causes of acquired epilepsy, i.e., posttraumatic epilepsy (PTE) [1] and disability [2,3,4]. PTE is one of the severest complications associated with TBI, occurring in 10–20% of patients [5,6]. The risk of unprovoked seizures is 10.2% after 2 years: 8% for mild TBI, 24% for moderate TBI, and 16.8% for severe TBI [6]. In most cases, PTE is of hippocampal origin (temporal lobe epilepsy) but may also have a neocortical origin—i.e., frontal, parietal, or occipital [7]. The relative risk of PTE is highest during the first two years after trauma, and 80% of all seizures develop during the first year [6,8].

Early posttraumatic seizures are mediated by acute primary and secondary mechanisms, while late unprovoked seizures are a manifestation of PTE. Acute seizures are a significant risk factor for PTE [9], as well as early focal slowing and epileptiform discharges on EEG [10]. During the late period after the development of the first unprovoked seizure, the probability of subsequent seizures is extremely high and, according to the ILAE criteria, allows for the diagnosis of epilepsy [11]. Other risk factors for the development of PTE include intracerebral hemorrhage, diffuse brain contusions, prolonged posttraumatic amnesia, fractures, and cortical–subcortical brain lesions [12].

Epileptogenesis is a process of functional changes in the brain’s neural networks that increases the likelihood of spontaneous recurrent seizures [13,14]. Mechanisms of epileptogenesis and biomarkers of PTE are actively studied in animal models. The gold-standard TBI model is lateral fluid percussion injury (LFPI) in rodents (rats or mice) [15,16,17,18,19]. LFPI in both rats [20] and mice [21] causes focal damage in the neocortex of the ipsilateral hemisphere (in the visual, sensorimotor, auditory, and parietal regions) [22]. LFPI can also induce secondary distant damage to the hippocampus and thalamus, as well as diffuse damage involving the brainstem and the contralateral hemisphere [23,24]. Both local and systemic mechanisms are involved in neuroinflammation and neurodegeneration after trauma [25,26]. 

According to the results of numerous studies of seizures and epileptogenesis [27,28,29], the spreading and maintenance of epileptiform activity involve two brain systems: the limbic and thalamocortical systems. The limbic system, consisting of the hippocampus and parahippocampal structures, is involved in the development of a specific form of epilepsy in rats that is similar to human medial temporal lobe epilepsy [28]. The thalamocortical system is associated with the development of generalized epilepsies, including absence epilepsy. In animal models, absence epilepsy is characterized by bilateral spike–wave discharges (SWDs) and behavioral arrests, corresponding to non-motor seizures [30,31,32]. Many groups pay attention to the damage to the hippocampus—the main epileptogenic structure in animal models of PTE; however, much less is known about changes in the thalamocortical system induced by focal injuries, including TBI.

Spontaneous SWDs have been observed in many rat strains, including in Sprague-Dawley rats after sham operation or TBI [33,34], in animal models of TLE [1], and in genetic models of absence epilepsy—e.g., GAERS (Genetic Absence Epilepsy Rats from Strasbourg) and Wag/Rij (Wistar Albino Glaxo Rats from Rijswijk). In genetic models of absence epilepsy, spontaneous SWDs at 7–12 Hz appear primarily during periods of immobility (e.g., behavioral arrests, freezing), constituting a pause in activity with open eyes and frozen posture. 

In our previous study, we found the presence of SWDs in “control” Sprague-Dawley rats, as well as after TBI or sham operation [35]. We showed an obvious heterogeneity of rats regarding the manifestation of SWDs. In 50% of rats, the average number of SWDs increased up to 20 times after TBI as compared to the background values before craniotomy. SWDs were accompanied by freezing behavior, associated with sleep phases, and the appearance of SWDs was correlated with increased astrogliosis in the neocortex. Thus, the appearance of SWDs and immature SWD-like events in the early posttraumatic period is an important feature of a TBI model and a possible marker of epileptogenesis. We hypothesized that the development of early posttraumatic SWDs involves the cortico-thalamocortical neuronal network. However, no confirmation of this hypothesis has been received in electrophysiological studies. Thus, the aim of this study was to assess the involvement of the thalamus in idiopathic and posttraumatic SWDs and their morphology in the neocortex of Sprague-Dawley rats.

## 2. Materials and Methods

All procedures were performed in accordance with the ARRIVE guidelines, the U.K. Animals (Scientific Procedures) Act 1986 and its associated guidelines, and the EU Directive 2010/63/EU for animal experiments. The Ethical Commission of the Institute of Higher Nervous Activity and Neurophysiology RAS approved our protocol (protocol # 2, 24 May 2017).

### 2.1. Animals

The experiments were carried out on 13 adult male Sprague-Dawley rats (supplied by Krolinfo breeding center, Moscow, Russia) aged 6 months at the beginning of the experiment, with mean body weight = 357 ± 8 g. Two groups of animals were used in the experiments: sham-operated animals (*n* = 5), and animals subjected to TBI (*n* = 8). The experimental animals were kept in individual plastic cages in the colony room with a 12 h light–dark cycle and free access to food and water.

### 2.2. Implantation of Electrodes

**Figure 1:**
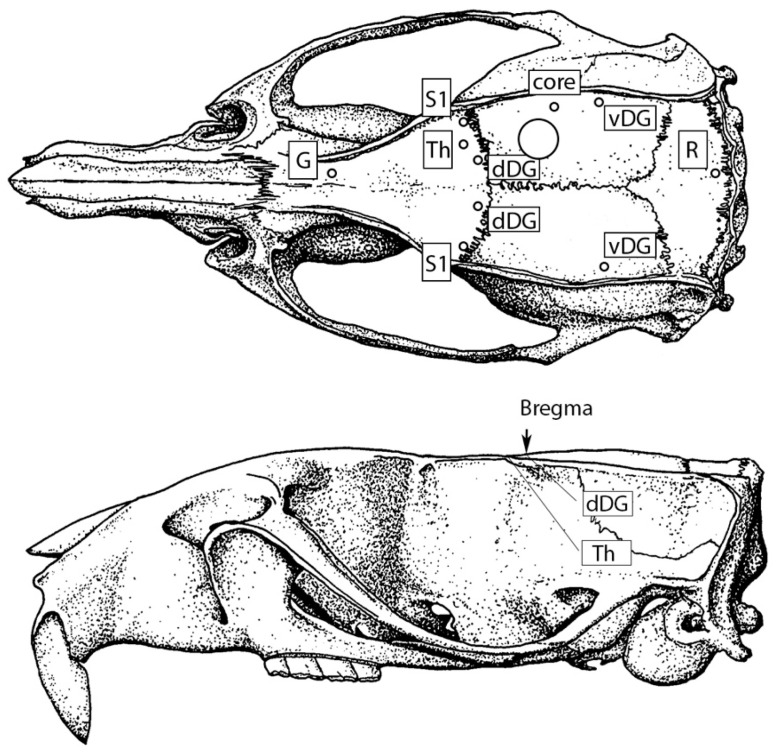
Scheme of depth electrode implantation and craniotomy site location: S1—primary sensory cortex, Th—thalamus, core—the area of TBI lesion in the neocortex, dDG and vDG—dorsal and ventral hippocampal dentate gyrus, respectively, G—ground electrode, R—reference electrode; the circle marks the area of craniotomy.

Isoflurane anesthesia (1–3%) was employed during all surgical procedures. The rats were mounted in a stereotaxic frame; eight nichrome depth electrodes (diameter 100 µm) for local field potential recordings were implanted bilaterally into the primary sensory cortex, perioral region (AP = 1, ML = ±5, H = −3.5), TBI core (AP = −4, ML = −5.5, H = −3)—the area of the maximal cortical lesion, according to our previous histological studies [25]—dorsal region (AP = 0, ML = ±2, H = −5.4, angle = 48°), ventral hippocampal dentate gyrus (AP = −6.3, ML = ±4.5, H = −4.3), and right ventral posteromedial nucleus of the thalamus (AP = 1, ML = 3, H = −7.5, angle = 37°) (Figure 1) A nichrome reference electrode was implanted in the occipital bone over the midline, and a nichrome ground electrode was implanted in the frontal bone over the midline. The electrodes were fixed with cyanoacrylate adhesive and dental cement, while the area of further craniotomy was kept intact.

### 2.3. TBI Modeling

On day 7 after electrode implantation, LFPI was performed. The rats were anesthetized with isoflurane, and a 4 mm diameter craniotomy was performed in the parietal bone (AP −3 mm, ML 3 mm) (Figure 1). Cyanoacrylate adhesive and dental cement were used to attach a female Luer-Loc connector to the craniotomy site. After removal from the stereotaxic frame and recovery from anesthesia, the animals were connected to the LFPI apparatus with an 80 cm long 3 mm polyvinyl chloride tube and underwent fluid percussion at 2.53 ± 0.11 atm. Rats from the sham-operated group went through all procedures applied to the TBI group except for the LFPI. The animals with unsuccessful surgery, damage to the dura mater, adhesive noticed on the dura mater, or improper TBI induction were excluded from the experiment.

### 2.4. In Vivo Electrophysiology

Video and local field potentials were synchronously recorded in home cages 24 h/day for 7 days before and 7 days after TBI induction. Local field potentials were recorded using a Wireless 2100-System-AO and headstages W2100-HS8-ES2-0.5 mA (Multichannel Systems, Reutlingen, Germany). The video was recorded with a DFK 33GX17 GigE camera. Recordings were performed in individual Plexiglas cages placed on a shielded rack (Open Science, Krasnogorsk, Russia). After the completion of the recordings, the animals were deeply anesthetized with chloral hydrate (395 mg/kg intraperitoneally) and were transcardially perfused with 4% paraformaldehyde in phosphate buffer (pH 7.4).

### 2.5. Detection of Epileptiform Activity

On day 6 of baseline recording (one day before TBI induction) and on day 7 after craniotomy, 24-h-long records were selected from each rat. Recordings were filtered in the EDF browser using a Butterworth bandpass filter at 1−30 Hz and divided into 10-s epochs. For each time point, 24 h (8640 epochs) per animal was analyzed. We marked SWDs and immature SWD-like events according to the following criteria: high-amplitude series of rhythmic spikes in the cortex, undoubtedly distinct from background activity with minimal duration 0.5 s (>4 consequent spikes), and with at least one evidently distinct wave after the spike. Usually, all spikes were directed up from baseline (negative), and negative deviation from the baseline was higher than positive deviation. The scoring was blinded for the persons performing it. In this study, we defined “immature” SWDs as not fully meeting the criteria of developed and “mature” SWDs (i.e., bilateral, lasting more than 1 s, with amplitude three times higher than the background [22]).

The morphology of 365 SWDs was analyzed. We considered predominantly spike–wave-like waveforms of the discharge when the presence of the well-defined wave was evident after more than three spikes in the discharge. Otherwise, we considered the event to be a discharge with a “predominantly spike” waveform (according to the initial criteria of SWDs, they should contain at least one wave). We also registered lateralization (unilateral or bilateral) and appearance in the thalamus. Involvement of the contralateral hemisphere was considered when a synchronous discharge of the same form—and with an increase in the amplitude of about 1.5 times compared to the background amplitude in the same channel—was evident.

All SWDs were divided into two groups: idiopathic SWDs (before craniotomy, *n* = 89 collected from 8 animals; 5 of the remaining implanted rats had no SWD-like events in the background) and posttraumatic SWDs (appeared after TBI in rats with low background SWD occurrence, *n* = 262). After TBI, we selected 262 posttraumatic SWDs from 2 animals with 1–2 SWDs per 24 h before craniotomy to avoid SWDs with mixed (idiopathic + posttraumatic) or unclear etiology. We did not use SWDs from the sham group after craniotomy in the analysis.

### 2.6. Statistical Methods

Statistical analysis was performed using Jamovi ver. 2.2.5.0. Graphics were made using GraphPad Prism 8. The numbers of SWDs in the TBI and sham groups were compared using the Mann–Whitney test; the numbers of SWDs at time points before and after craniotomy were compared using the Wilcoxon test. The proportions of SWD characteristics were compared using Fisher’s exact test; for coexistence of two parameters, odds ratios with 95% confidence intervals were calculated. Segregation by etiology based on specific features of SWDs was performed using binominal logistic regression followed by receiver operating characteristic (ROC) analysis. Predictions of thalamus involvement and etiology based on specific features of SWDs were assessed with binominal logistic regression followed by ROC analysis. All data are presented as the mean ± SEM (standard error of the mean).

## 3. Results

### 3.1. SWD Characteristics and Time Course during the Experiment

**Figure 2:**
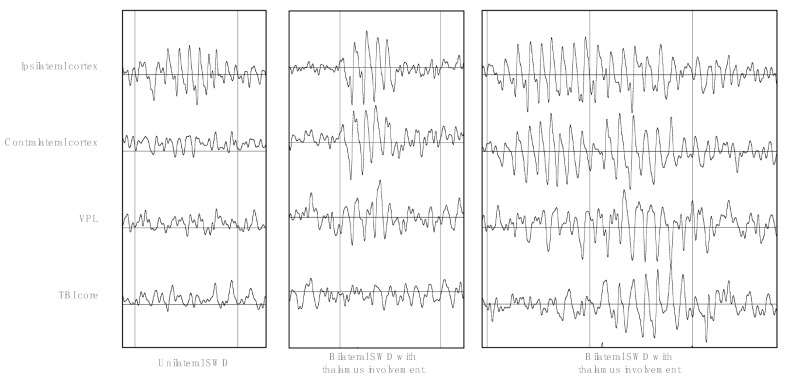
Morphology of SWDs: Examples of unilateral SWD (left panel), bilateral wave-like discharge with thalamus involvement (middle panel), and bilateral spike–wave discharge with thalamus involvement and spreading over the ipsilateral cortex (right panel).

Mortality in the experiment was 37.5% (3 of 8 rats with TBI). The average number of SWDs and immature SWD-like events was 7 ± 5 SWD/day before craniotomy (89 discharges, *n* = 12 rats) and 31 ± 19 SWD/day after craniotomy (276, discharges, *n* = 9 rats), and the results did not differ between the TBI and sham groups before (*p* = 0.083) and after craniotomy (*p* = 0.893). Additionally, the number of SWDs did not change after craniotomy as compared to the background level (*p* = 0.098 in the sham group, *p* = 0.371 in the TBI group), most likely due to the presence of idiopathic SWDs and the rather small numbers of animals in the groups. SWDs might appear ipsilaterally or spread over both hemispheres, and they might involve the thalamic ventral posterolateral nucleus (Figure 2).

### 3.2. Waveform, Lateralization, and Thalamus Involvement

**Figure 3:**
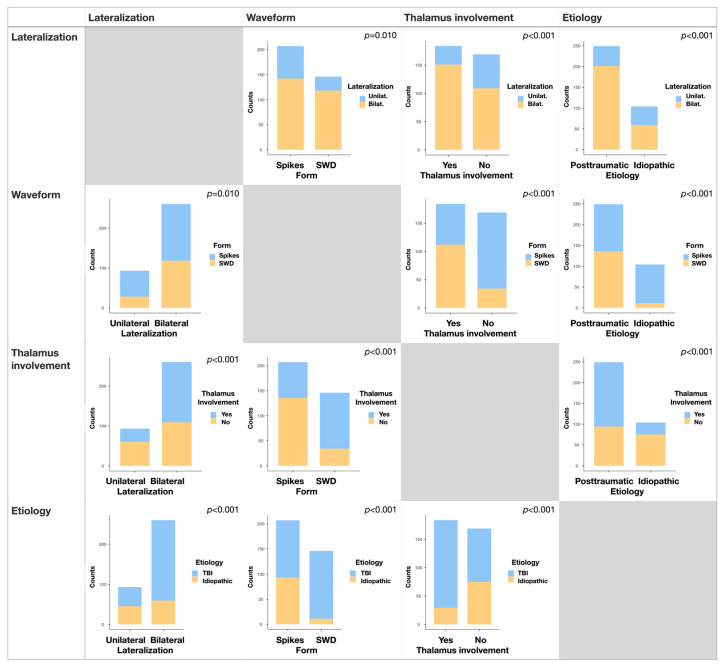
Specific features of idiopathic and posttraumatic SWDs compared with Fisher’s exact test.

**Figure 4:**
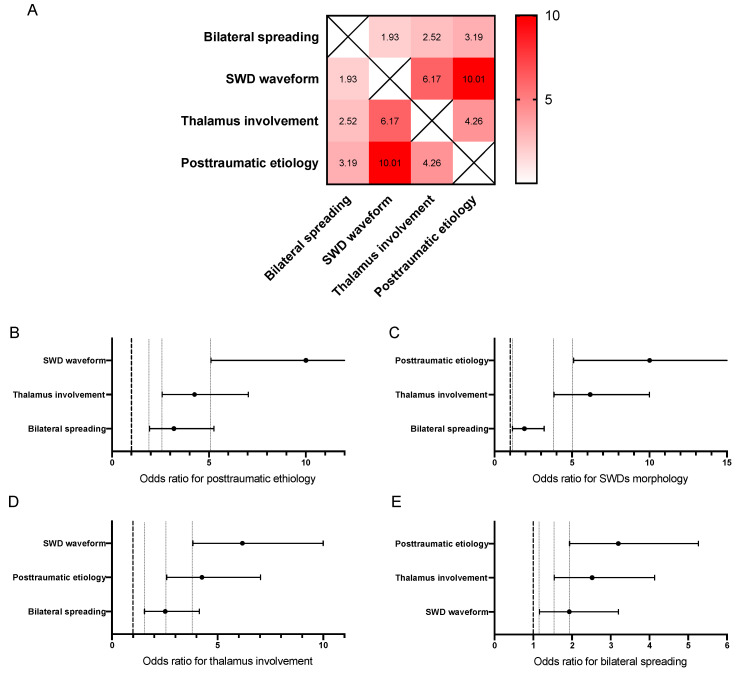
Odds ratios between specific features of SWDs: (**A**) Heatmap of odds ratios. (**B**–**E**) Odds ratios between SWDs’ characteristics (OR with 95% CI).

To confirm the hypothesis of thalamus involvement in SWDs and early immature SWD-like generation in Sprague-Dawley rats, we compared the appearance of SWDs in the thalamus with the waveform and lateralization (Figure 3) for all selected SWDs (*n* = 365). We found that occurrence of SWDs in the thalamus determined the spike–wave form of SWDs and bilateral lateralization in the neocortex. SWDs in the neocortex were recorded significantly more frequently in both hemispheres than unilateral ones if SWDs were simultaneously present in the thalamus (*p* < 0.001, Fisher’s exact test). The shape of the discharges depended on the presence of SWDs in the thalamus; in this situation, a spike–wave form of SWD was evident more often than a predominantly spike form without a subsequent wave (*p* < 0.001, Fisher’s exact test). We also calculated odds ratios (ORs) to compare the relationships between SWD characteristics (Figure 4). If we detected involvement of the thalamus in SWD generation, the OR of bilateral spreading in the neocortex was 2.5 [95% CI 1.5, 4.1], and the OR of the SWD waveform was 3.8 [6.2, 10.0].

### 3.3. Posttraumatic vs. Idiopathic Etiology

We also compared SWD characteristics depending on their etiology (Figure 3). Posttraumatic SWDs (i.e., appearing after TBI in rats with low background SWD occurrence, *n* = 262), as compared to idiopathic SWDs (i.e., all SWDs before craniotomy, *n* = 89), were characterized by bilateral spreading (*p* < 0.001), a spike–wave form (*p* < 0.001), and thalamus involvement (*p* < 0.001). For posttraumatic SWDs, the OR of bilateral spreading in the neocortex was 3.2 [1.9, 5.2], the OR for involvement of the thalamus was 4.3 [2.6, 7.0], and the OR of SWDs with a clear spike–wave form was 10.0 [5.1, 19.6]. Thus, the probability of SWDs with a clear spike–wave form having a posttraumatic etiology was 10-fold higher than that of an idiopathic etiology (Figure 4). We can also note that these characteristics of posttraumatic SWDs correspond to more “mature” SWDs as compared with immature SWD-like idiopathic discharges.

### 3.4. Segregation of SWDs by Etiology

**Figure 5:**
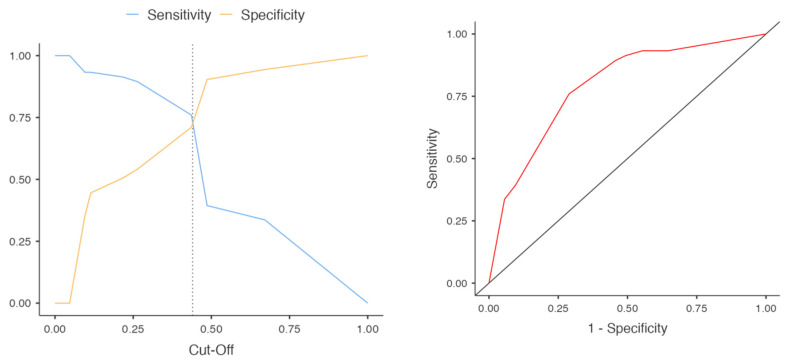
Cutoff plot and ROC curve for the prediction of SWDs’ etiology based on their lateralization, SWD morphology, and the involvement of the thalamus.

Based on the assumption that the form of SWDs and the involvement of the thalamus can be different for posttraumatic and idiopathic SWDs, we performed binary logistic regression. We showed that all three indices significantly segregated SWDs by their etiology: lateralization (*p* < 0.001), SWD morphology (*p* < 0.001), and the involvement of the thalamus (*p* < 0.01). The cutoff plot and ROC curves are presented in Figure 5. The accuracy of the segregation was 0.754, the specificity was 0.904, the sensitivity was 0.394, and the AUC (area under the ROC curve) was 0.791. The model had low sensitivity but high specificity for posttraumatic SWDs. Segregation of SWDs by their etiology is of particular importance in frequent situations when background recordings before craniotomy are not available. At the start, the detection of posttraumatic SWDs with high specificity is more important than the detection of all SWDs with high sensitivity (if the latter goal is at all attainable).

## 4. Discussion

In this study, we compared the morphology and lateralization of idiopathic and posttraumatic SWDs and immature SWD-like events, as well as the involvement of the thalamus in their generation. We found a relationship between the presence of SWDs in the thalamus and their more “mature” characteristics, i.e., spike–wave form and bilateral spreading over the neocortex. We also showed that SWD characteristics can be used to segregate idiopathic and posttraumatic discharges with high specificity. Posttraumatic SWDs had a more mature morphology compared to idiopathic SWD-like events in rats before craniotomy, i.e., spike–wave form, bilateral spreading, and thalamic involvement. This confirms that the generation of posttraumatic SWDs involves the cortico-thalamocortical neuronal network. 

In this study, we used LFPI—an approach that is widely used in rodent TBI models [16]. Trauma is inflicted via pulsed short pressure of a fluid on the intact dura mater [36]. The craniotomy site can be located along the median line (median fluid percussion) or laterally from the median line (lateral fluid percussion) [37]. LFPI can induce the development of cognitive impairment and increased anxiety in rats, making this model highly clinically relevant and translatable [38]. Primary damage occurs directly as a result of the initial impact and entails direct mechanical damage to the brain, i.e., rupture of cell membranes, mechanical disruption of the blood–brain barrier, and the release of albumin and other blood components into the intercellular space. The mechanisms of secondary damage are also initiated at the moment of injury but continue for a longer time through molecular, cellular, and physiological processes, i.e., changes in the blood–brain barrier’s permeability, excitotoxicity, oxidative stress, inflammation, mitochondrial dysfunction, degeneration, and death of inhibitory neurons [39]. Accumulation of excitatory neurotransmitters and excessive activation of their receptors induces subsequent activation of intracellular death cascades. Epileptiform activity may exacerbate excitotoxic neurodegeneration and trigger the epileptogenic process.

Epileptogenesis based on alterations of neural networks creates a source for spontaneous recurrent seizures [13]. The development of epileptic networks is maintained by exciting positive feedback—a specific feature of such networks formed as a result of the inhibitory circuit loss [40]. Another mechanism of positive feedback development after TBI is the emergence of new synaptic connections between surviving neurons [41,42,43]. Axon sprouting and neuronal cell are is the main structural pathological processes underlying epileptogenesis [44].

Revealing biomarkers of epileptogenesis is vital, since they are a tool to predict the development of epilepsy and can also serve as indicators of its progression [45,46]. The appearance of early epileptiform activity in electrographic recordings is regarded as a promising predictor of posttraumatic epilepsy. The mechanisms of epileptiform activity and seizures are different in the early and late posttraumatic periods: early epileptiform activity is associated with primary and early secondary brain damage, while late epileptiform activity is a marker of PTE. Late unprovoked seizures, according to reports from many laboratories, occur in 20–40% of rats after TBI [47,48]. The latent period of late seizures—including focal cortical seizures and seizures of subcortical origin—is 4–6 weeks [49,50], and the proportion of focal seizures with spreading increases (observations from 2 to 7 weeks after TBI).

SWDs have been recorded in electrocorticograms weeks and months after fluid percussion injury in Sprague-Dawley rats [33]. The number and duration of SWD events increased with age, and the SWD burst length parameters followed very similar age-related patterns and were not significantly different from those of the uninjured controls. Approximately 19% of naïve female 2-month-old Sprague-Dawley rats exhibited SWDs spontaneously during behavioral arrest, while male Sprague-Dawley rats exhibited SWDs after the age of 3 months [51]. Nevertheless, it remains unknown how TBI affects SWD generation in animals demonstrating idiopathic SWDs. In our previous study on Sprague-Dawley rats, we found SWDs both in the background and after TBI or sham operation. In half of the rats, TBI induced a dramatic increase in the occurrence of SWDs compared to before craniotomy [35], indicating the presence of SWDs triggered by TBI in rats. Associations of SWDs with freezing behavior, sleep phases, and cortical astroglial density in the neocortex were also described. Early immature SWDs may serve as a potential biomarker of late seizures and epileptogenesis; however, differentiation of posttraumatic and idiopathic SWDs in the experimental research, though vital, remains an unsolved issue. In this study, our analysis also included discharges that did not fully meet the criteria of developed, “mature” SWD (the latter should be bilateral and last more than 1 s, with amplitude three times higher than the background for the discrimination of posttraumatic and spontaneously generated (idiopathic) discharges [32]). This approach allowed us to show that posttraumatic SWD characteristics are more “mature” as compared to idiopathic SWDs; they were characterized by spike–wave form, bilateral spreading, and thalamic involvement.

SWD mechanisms are well described in genetic animal models of absence epilepsy. In these animals, SWDs are thalamocortical oscillations that are considered to be EEG correlates of absence seizures. In GAERS and Wag/Rij rat strains, SWDs are a reliable marker of disease, and changes in the occurrence of SWDs may serve as a marker of epilepsy’s progression. Typical SWDs—predominantly bifrontal and accompanied by freezing—have been described in WAG/Rij rats [32]. According to the “cortico-reticular” concept, sleep-related oscillations may give rise to the generation of SWDs; however, there is convincing evidence of the “cortical” concept, claiming a neocortical focus of SWD generation [30]. SWDs were shown to be triggered in the somatosensory cortex and supported by a cortico-thalamocortical neuronal network [30], with the thalamus presumably playing a secondary role in SWD generation. The role of thalamic nuclei in genetic generalized epilepsy were reviewed recently [52]. The classical three-compartment hypothesis of SWD generation includes (1) the perioral region of the somatosensory cortex (S1), (2) glutamatergic thalamocortical (TC) relay neurons of the ventrobasal thalamus, and (3) GABAergic neurons of the reticular thalamic nucleus (RTn). According to this model, the epileptic onset zone is located in layers V and VI of S1. The firing of thalamocortical neurons provides rhythmic excitation to the TC relay neurons and via collaterals to the inhibitory RTn. RTn neurons send axons to the TC neurons, and their hyperpolarization promotes burst firing consisting of rhythmic excitation and inhibition. Recently, network mechanisms of the spike and wave components of SWD generation were shown. Using local pharmacological inhibition of the centromedian thalamus, an involvement of thalamic inputs in the induction of the spike component in cortical microcircuits was demonstrated, while the wave component’s generation was associated with intracortical microcircuits [53,54]. Another hypothesis implies that SWDs facilitate the processing of sensory inputs that are acquired during SWDs [55,56]. Different neuronal seizure-associated activities were demonstrated using functional magnetic resonance imaging (MRI) and neuronal activity recordings in GAERS rats [57]. Changes occurred 40–60 s prior to the seizure and included decreased overall firing rate and increased rhythmicity in distinct functional groups of cortical and thalamic neurons.

Several studies have also revealed a link between damage to the thalamus and PTE. Reorganization of parvalbumin-positive interneuron terminals in the ventral thalamic nuclei was shown after TBI in rats [58]. In addition, the severity and evolution of thalamic pathology on MRI predicted late posttraumatic seizures in rats. The best multivariable logistic regression model based on MRI on days 2, 7, and 21 resulted in a cross-validated AUROC of 0.78 [59]. Thus, pathology in the ventral thalamic nuclei predicts PTE with moderate sensitivity and high specificity. Thalamic involvement in the development of PTE has translational potential. Chronic blood–brain barrier dysfunction was shown in the thalami of patients with PTE, including albumin accumulation, iron deposits, calcifications, and the presence of the markers of neuroinflammation, such as astrocytes, microglia, and monocytes [60]. Microglial activation in the thalamus was shown using positron emission tomography/computed tomography in patients after TBI, and it was associated with more severe cognitive impairment [61].

Taking into account (1) the “cortical” hypothesis of SWD generation in animal models of absence epilepsy, (2) the correlation of the area of astrogliosis with posttraumatic SWD occurrence, and (3) the prediction of PTE based on morphological changes in the thalamic nuclei, it could be assumed that damage to the somatosensory cortex in rat models of TBI may trigger the progression of the immature SWD-like oscillations to more “mature” SWD-like phenomena with a spike–wave form and bilateral spreading via the thalamocortical neuronal network. The occurrence of posttraumatic SWDs and/or their characteristics could be used as possible markers of PTE development.

## 5. Conclusions

In the present study, we showed that specific features of SWDs allow for the distinction between idiopathic and posttraumatic discharges with high specificity. We also described a relationship between the occurrence of SWDs in the thalamus and their more “mature” characteristics, i.e., spike–wave form and bilateral spreading over the neocortex. Our results support the hypothesis that the formation of posttraumatic SWDs involves the cortico-thalamocortical neuronal network(s). These results are important for extending knowledge on the mechanisms underlying the development and occurrence of epileptiform activity and epileptogenesis.

## 6. Limitations

The number of rats (*n* = 13) is a limitation of this study, since analysis based on a larger number of SWDs may improve the segregation of idiopathic and posttraumatic SWDs. More precise analysis of idiopathic and posttraumatic immature SWDs in Sprague-Dawley rats could reveal additional parameters that also may improve discrimination (e.g., time–frequency characteristics, which were not included in the analysis). Another limitation may be the lack of data on the remote posttraumatic period in this experiment; the progression and role of immature early SWDs in the development of PTE still need to be studied further.

## Data Availability

Data are available upon reasonable request.

## Abbreviations

AUC	Area under the ROC curve
LFPI	Lateral fluid percussion injury
MRI	Magnetic resonance imaging
OR	Odds ratio
PTE	Posttraumatic epilepsy
ROC	Receiver operating characteristic
RTn	Reticular thalamic nucleus
S1	Somatosensory cortex
SWD	Spike–wave discharge
TBI	Traumatic brain injury
TC	Thalamocortical
